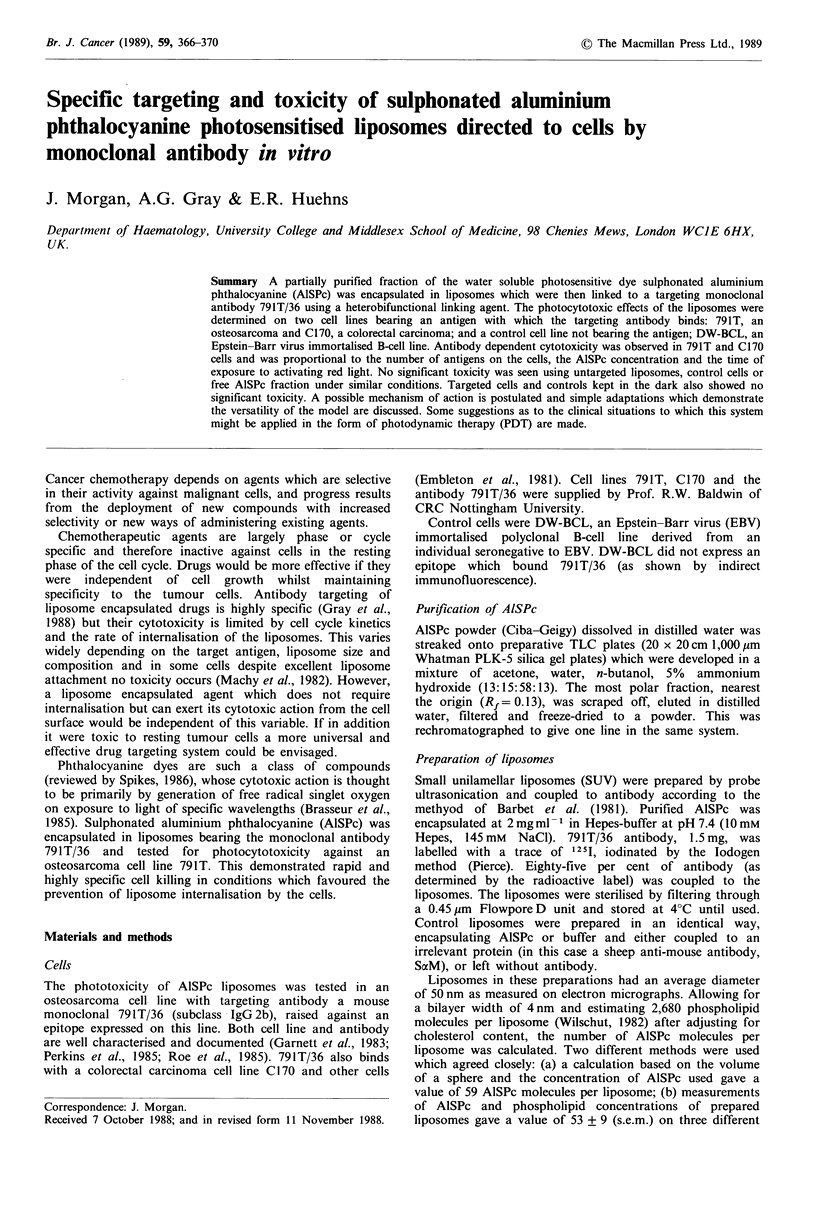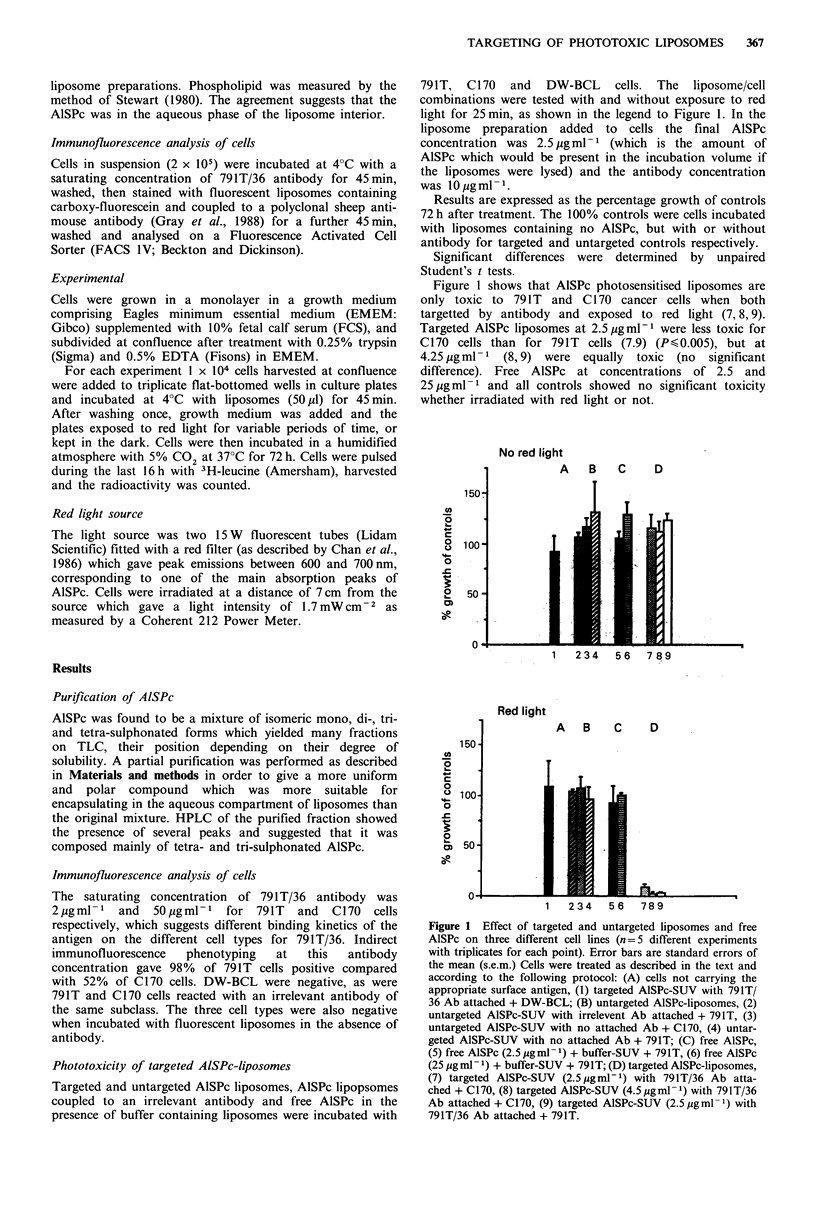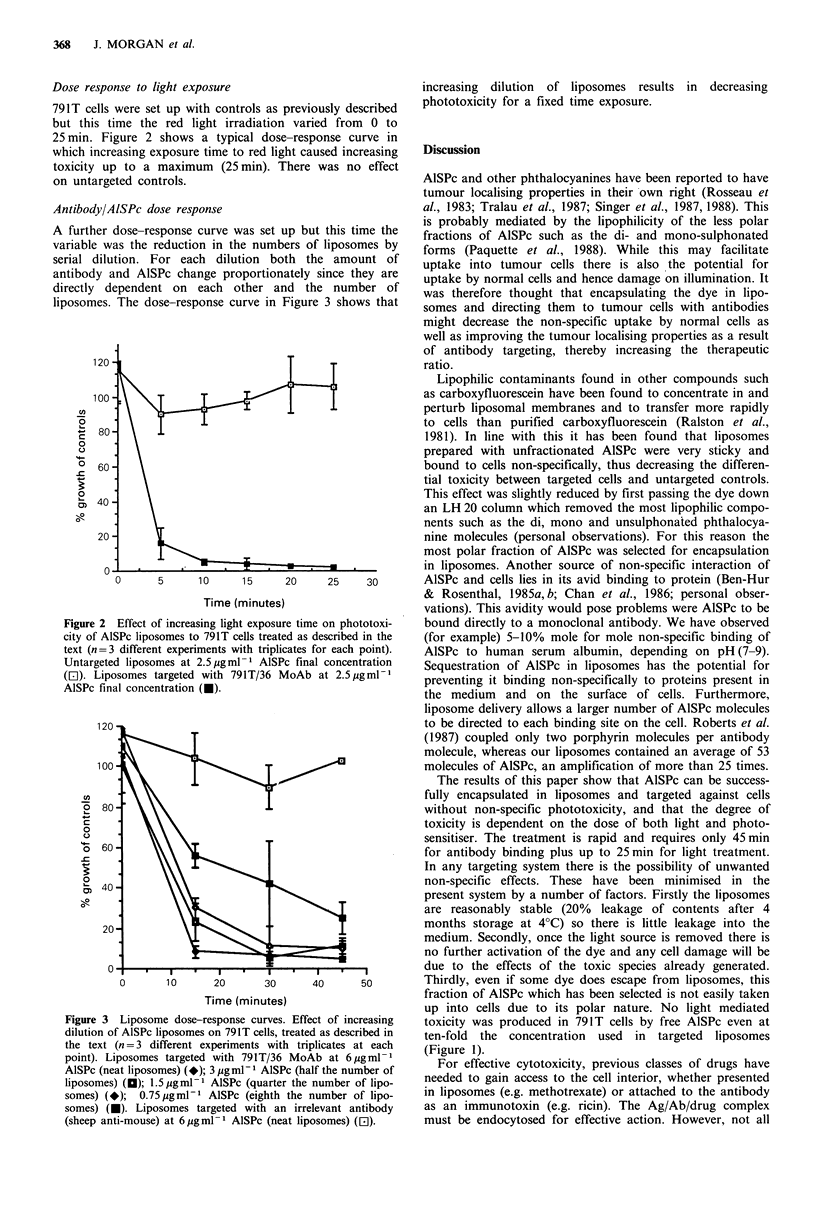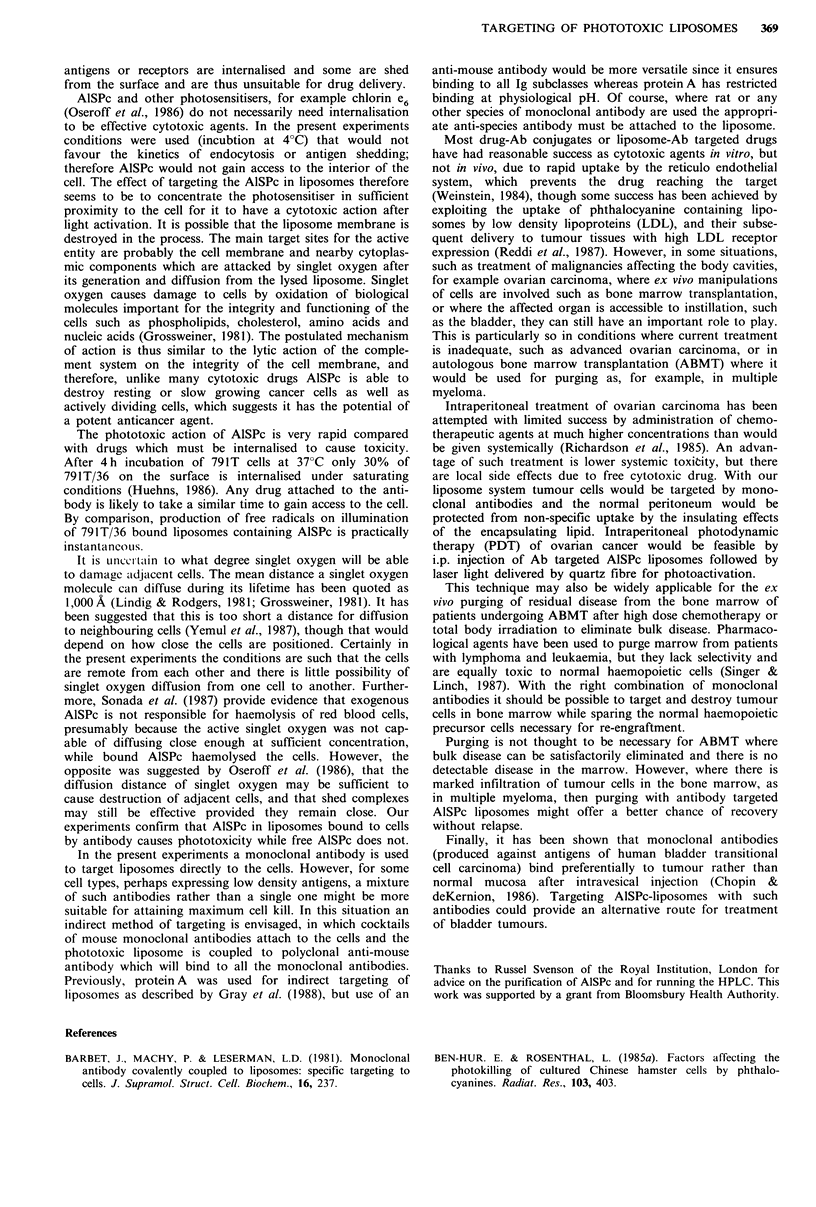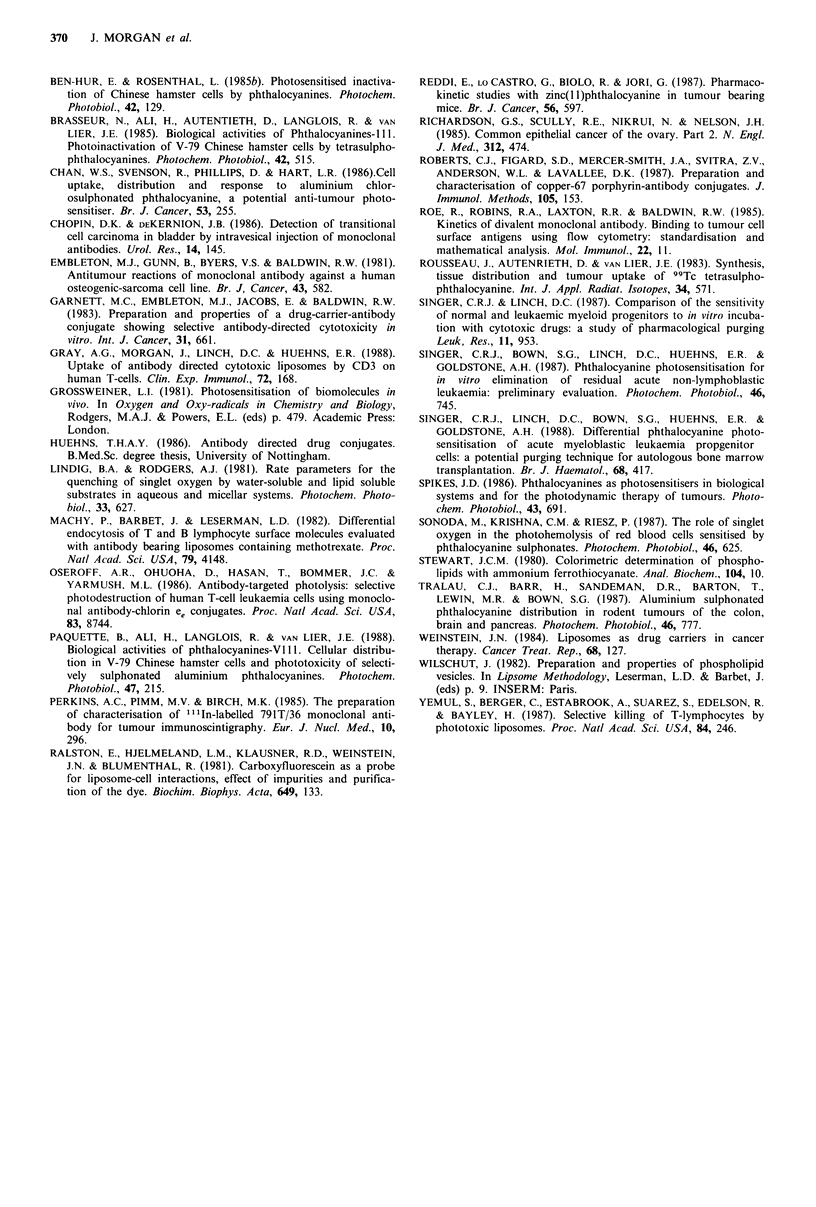# Specific targeting and toxicity of sulphonated aluminium phthalocyanine photosensitised liposomes directed to cells by monoclonal antibody in vitro.

**DOI:** 10.1038/bjc.1989.73

**Published:** 1989-03

**Authors:** J. Morgan, A. G. Gray, E. R. Huehns

**Affiliations:** Department of Haematology, University College, Middlesex School of Medicine, London, UK.

## Abstract

A partially purified fraction of the water soluble photosensitive dye sulphonated aluminium phthalocyanine (AlSPc) was encapsulated in liposomes which were then linked to a targeting monoclonal antibody 791T/36 using a heterobifunctional linking agent. The photocytotoxic effects of the liposomes were determined on two cell lines bearing an antigen with which the targeting antibody binds: 791T, an osteosarcoma and C170, a colorectal carcinoma; and a control cell line not bearing the antigen; DW-BCL, an Epstein-Barr virus immortalised B-cell line. Antibody dependent cytotoxicity was observed in 791T and C170 cells and was proportional to the number of antigens on the cells, the AlSPc concentration and the time of exposure to activating red light. No significant toxicity was seen using untargeted liposomes, control cells or free AlSPc fraction under similar conditions. Targeted cells and controls kept in the dark also showed no significant toxicity. A possible mechanism of action is postulated and simple adaptations which demonstrate the versatility of the model are discussed. Some suggestions as to the clinical situations to which this system might be applied in the form of photodynamic therapy (PDT) are made.


Br. J. Cancer (1989), 59, 366–370 © The Macmillan Press Ltd., 1989

### Specific targeting and toxicity of sulphonated aluminium phthalocyanine photosensitised liposomes directed to cells by monoclonal antibody *in vitro*

J. Morgan, A.G. Gray & E.R. Huehns

*Department of Haematology, University College and Middlesex School of Medicine, 98 Chenies Mews, London WC1E 6HX, UK.*

**Summary** A partially purified fraction of the water soluble photosensitive dye sulphonated aluminium phthalocyanine (AlSPc) was encapsulated in liposomes which were then linked to a targeting monoclonal antibody 791T/36 using a heterobifunctional linking agent. The photocytotoxic effects of the liposomes were determined on two cell lines bearing an antigen with which the targeting antibody binds: 791T, an osteosarcoma and C170, a colorectal carcinoma; and a control cell line not bearing the antigen; DW-BCL, an Epstein–Barr virus immortalised B-cell line. Antibody dependent cytotoxicity was observed in 791T and C170 cells and was proportional to the number of antigens on the cells, the AlSPc concentration and the time of exposure to activating red light. No significant toxicity was seen using untargeted liposomes, control cells or free AlSPc fraction under similar conditions. Targeted cells and controls kept in the dark also showed no significant toxicity. A possible mechanism of action is postulated and simple adaptations which demonstrate the versatility of the model are discussed. Some suggestions as to the clinical situations to which this system might be applied in the form of photodynamic therapy (PDT) are made.

Cancer chemotherapy depends on agents which are selective in their activity against malignant cells, and progress results from the deployment of new compounds with increased selectivity or new ways of administering existing agents.

Chemotherapeutic agents are largely phase or cycle specific and therefore inactive against cells in the resting phase of the cell cycle. Drugs would be more effective if they were independent of cell growth whilst maintaining specificity to the tumour cells. Antibody targeting of liposome encapsulated drugs is highly specific (Gray *et al.*, 1988) but their cytotoxicity is limited by cell cycle kinetics and the rate of internalisation of the liposomes. This varies widely depending on the target antigen, liposome size and composition and in some cells despite excellent liposome attachment no toxicity occurs (Machy *et al.*, 1982). However, a liposome encapsulated agent which does not require internalisation but can exert its cytotoxic action from the cell surface would be independent of this variable. If in addition it were toxic to resting tumour cells a more universal and effective drug targeting system could be envisaged.

Phthalocyanine dyes are such a class of compounds (reviewed by Spikes, 1986), whose cytotoxic action is thought to be primarily by generation of free radical singlet oxygen on exposure to light of specific wavelengths (Brasseur *et al.*, 1985). Sulphonated aluminium phthalocyanine (AlSPc) was encapsulated in liposomes bearing the monoclonal antibody 791T/36 and tested for photocytotoxicity against an osteosarcoma cell line 791T. This demonstrated rapid and highly specific cell killing in conditions which favoured the prevention of liposome internalisation by the cells.

### Materials and methods

### *Cells*

The phototoxicity of AlSPc liposomes was tested in an osteosarcoma cell line with targeting antibody a mouse monoclonal 791T/36 (subclass IgG 2b), raised against an epitope expressed on this line. Both cell line and antibody are well characterised and documented (Garnett *et al.*, 1983; Perkins *et al.*, 1985; Roe *et al.*, 1985). 791T/36 also binds with a colorectal carcinoma cell line C170 and other cells (Embleton *et al.*, 1981). Cell lines 791T, C170 and the antibody 791T/36 were supplied by Prof. R.W. Baldwin of CRC Nottingham University.

Control cells were DW-BCL, an Epstein–Barr virus (EBV) immortalised polyclonal B-cell line derived from an individual seronegative to EBV. DW-BCL did not express an epitope which bound 791T/36 (as shown by indirect immunofluorescence).

### *Purification of AlSPc*

AlSPc powder (Ciba–Geigy) dissolved in distilled water was streaked onto preparative TLC plates (20 × 20 cm 1,000 μm Whatman PLK-5 silica gel plates) which were developed in a mixture of acetone, water, *n*-butanol, 5% ammonium hydroxide (13:15:58:13). The most polar fraction, nearest the origin ($R_f = 0.13$), was scraped off, eluted in distilled water, filtered and freeze-dried to a powder. This was rechromatographed to give one line in the same system.

### *Preparation of liposomes*

Small unilamellar liposomes (SUV) were prepared by probe ultrasonication and coupled to antibody according to the methyod of Barbet *et al.* (1981). Purified AlSPc was encapsulated at 2 mg ml$^{-1}$ in Hepes-buffer at pH 7.4 (10 mM Hepes, 145 mM NaCl). 791T/36 antibody, 1.5 mg, was labelled with a trace of $^{125}$I, iodinated by the Iodogen method (Pierce). Eighty-five per cent of antibody (as determined by the radioactive label) was coupled to the liposomes. The liposomes were sterilised by filtering through a 0.45 μm Flowpore D unit and stored at 4°C until used. Control liposomes were prepared in an identical way, encapsulating AlSPc or buffer and either coupled to an irrelevant protein (in this case a sheep anti-mouse antibody, SαM), or left without antibody.

Liposomes in these preparations had an average diameter of 50 nm as measured on electron micrographs. Allowing for a bilayer width of 4 nm and estimating 2,680 phospholipid molecules per liposome (Wilschut, 1982) after adjusting for cholesterol content, the number of AlSPc molecules per liposome was calculated. Two different methods were used which agreed closely: (a) a calculation based on the volume of a sphere and the concentration of AlSPc used gave a value of 59 AlSPc molecules per liposome; (b) measurements of AlSPc and phospholipid concentrations of prepared liposomes gave a value of 53 ± 9 (s.e.m.) on three different

Correspondence: J. Morgan.
Received 7 October 1988; and in revised form 11 November 1988.

liposome preparations. Phospholipid was measured by the method of Stewart (1980). The agreement suggests that the AlSPc was in the aqueous phase of the liposome interior.

*Immunofluorescence analysis of cells*

Cells in suspension (2 × $10^5$) were incubated at 4°C with a saturating concentration of 791T/36 antibody for 45 min, washed, then stained with fluorescent liposomes containing carboxy-fluorescein and coupled to a polyclonal sheep anti-mouse antibody (Gray *et al.*, 1988) for a further 45 min, washed and analysed on a Fluorescence Activated Cell Sorter (FACS 1V; Beckton and Dickinson).

*Experimental*

Cells were grown in a monolayer in a growth medium comprising Eagles minimum essential medium (EMEM: Gibco) supplemented with 10% fetal calf serum (FCS), and subdivided at confluence after treatment with 0.25% trypsin (Sigma) and 0.5% EDTA (Fisons) in EMEM.

For each experiment 1 × $10^4$ cells harvested at confluence were added to triplicate flat-bottomed wells in culture plates and incubated at 4°C with liposomes (50 µl) for 45 min. After washing once, growth medium was added and the plates exposed to red light for variable periods of time, or kept in the dark. Cells were then incubated in a humidified atmosphere with 5% $CO_2$ at 37°C for 72 h. Cells were pulsed during the last 16 h with $^3$H-leucine (Amersham), harvested and the radioactivity was counted.

*Red light source*

The light source was two 15 W fluorescent tubes (Lidam Scientific) fitted with a red filter (as described by Chan *et al.*, 1986) which gave peak emissions between 600 and 700 nm, corresponding to one of the main absorption peaks of AlSPc. Cells were irradiated at a distance of 7 cm from the source which gave a light intensity of 1.7 mW $cm^{-2}$ as measured by a Coherent 212 Power Meter.

### Results

*Purification of AlSPc*

AlSPc was found to be a mixture of isomeric mono, di-, tri- and tetra-sulphonated forms which yielded many fractions on TLC, their position depending on their degree of solubility. A partial purification was performed as described in **Materials and methods** in order to give a more uniform and polar compound which was more suitable for encapsulating in the aqueous compartment of liposomes than the original mixture. HPLC of the purified fraction showed the presence of several peaks and suggested that it was composed mainly of tetra- and tri-sulphonated AlSPc.

*Immunofluorescence analysis of cells*

The saturating concentration of 791T/36 antibody was 2 µg $ml^{-1}$ and 50 µg $ml^{-1}$ for 791T and C170 cells respectively, which suggests different binding kinetics of the antigen on the different cell types for 791T/36. Indirect immunofluorescence phenotyping at this antibody concentration gave 98% of 791T cells positive compared with 52% of C170 cells. DW-BCL were negative, as were 791T and C170 cells reacted with an irrelevant antibody of the same subclass. The three cell types were also negative when incubated with fluorescent liposomes in the absence of antibody.

*Phototoxicity of targeted AlSPc-liposomes*

Targeted and untargeted AlSPc liposomes, AlSPc lipopsomes coupled to an irrelevant antibody and free AlSPc in the presence of buffer containing liposomes were incubated with 791T, C170 and DW-BCL cells. The liposome/cell combinations were tested with and without exposure to red light for 25 min, as shown in the legend to Figure 1. In the liposome preparation added to cells the final AlSPc concentration was 2.5 µg $ml^{-1}$ (which is the amount of AlSPc which would be present in the incubation volume if the liposomes were lysed) and the antibody concentration was 10 µg $ml^{-1}$.

Results are expressed as the percentage growth of controls 72 h after treatment. The 100% controls were cells incubated with liposomes containing no AlSPc, but with or without antibody for targeted and untargeted controls respectively.

Significant differences were determined by unpaired Student's *t* tests.

Figure 1 shows that AlSPc photosensitised liposomes are only toxic to 791T and C170 cancer cells when both targetted by antibody and exposed to red light (7, 8, 9). Targeted AlSPc liposomes at 2.5 µg $ml^{-1}$ were less toxic for C170 cells than for 791T cells (7.9) ($P \leq 0.005$), but at 4.25 µg $ml^{-1}$ (8, 9) were equally toxic (no significant difference). Free AlSPc at concentrations of 2.5 and 25 µg $ml^{-1}$ and all controls showed no significant toxicity whether irradiated with red light or not.

**Figure 1** Effect of targeted and untargeted liposomes and free AlSPc on three different cell lines ($n = 5$ different experiments with triplicates for each point). Error bars are standard errors of the mean (s.e.m.) Cells were treated as described in the text and according to the following protocol: (A) cells not carrying the appropriate surface antigen, (1) targeted AlSPc-SUV with 791T/36 Ab attached + DW-BCL; (B) untargeted AlSPc-liposomes, (2) untargeted AlSPc-SUV with irrelevent Ab attached + 791T, (3) untargeted AlSPc-SUV with no attached Ab + C170, (4) untargeted AlSPc-SUV with no attached Ab + 791T; (C) free AlSPc, (5) free AlSPc (2.5 µg $ml^{-1}$) + buffer-SUV + 791T, (6) free AlSPc (25 µg $ml^{-1}$) + buffer-SUV + 791T; (D) targeted AlSPc-liposomes, (7) targeted AlSPc-SUV (2.5 µg $ml^{-1}$) with 791T/36 Ab attached + C170, (8) targeted AlSPc-SUV (4.5 µg $ml^{-1}$) with 791T/36 Ab attached + C170, (9) targeted AlSPc-SUV (2.5 µg $ml^{-1}$) with 791T/36 Ab attached + 791T.

*Dose response to light exposure*

791T cells were set up with controls as previously described but this time the red light irradiation varied from 0 to 25 min. Figure 2 shows a typical dose–response curve in which increasing exposure time to red light caused increasing toxicity up to a maximum (25 min). There was no effect on untargeted controls.

*Antibody/AlSPc dose response*

A further dose–response curve was set up but this time the variable was the reduction in the numbers of liposomes by serial dilution. For each dilution both the amount of antibody and AlSPc change proportionately since they are directly dependent on each other and the number of liposomes. The dose–response curve in Figure 3 shows that

**Figure 2** Effect of increasing light exposure time on phototoxicity of AlSPc liposomes to 791T cells treated as described in the text ($n = 3$ different experiments with triplicates for each point). Untargeted liposomes at 2.5 μg ml$^{-1}$ AlSPc final concentration (□). Liposomes targeted with 791T/36 MoAb at 2.5 μg ml$^{-1}$ AlSPc final concentration (■).

**Figure 3** Liposome dose–response curves. Effect of increasing dilution of AlSPc liposomes on 791T cells, treated as described in the text ($n = 3$ different experiments with triplicates at each point). Liposomes targeted with 791T/36 MoAb at 6 μg ml$^{-1}$ AlSPc (neat liposomes) (◆); 3 μg ml$^{-1}$ AlSPc (half the number of liposomes) (▣); 1.5 μg ml$^{-1}$ AlSPc (quarter the number of liposomes) (◆); 0.75 μg ml$^{-1}$ AlSPc (eighth the number of liposomes) (■). Liposomes targeted with an irrelevant antibody (sheep anti-mouse) at 6 μg ml$^{-1}$ AlSPc (neat liposomes) (□).

increasing dilution of liposomes results in decreasing phototoxicity for a fixed time exposure.

### Discussion

AlSPc and other phthalocyanines have been reported to have tumour localising properties in their own right (Rosseau et al., 1983; Tralau et al., 1987; Singer et al., 1987, 1988). This is probably mediated by the lipophilicity of the less polar fractions of AlSPc such as the di- and mono-sulphonated forms (Paquette et al., 1988). While this may facilitate uptake into tumour cells there is also the potential for uptake by normal cells and hence damage on illumination. It was therefore thought that encapsulating the dye in liposomes and directing them to tumour cells with antibodies might decrease the non-specific uptake by normal cells as well as improving the tumour localising properties as a result of antibody targeting, thereby increasing the therapeutic ratio.

Lipophilic contaminants found in other compounds such as carboxyfluorescein have been found to concentrate in and perturb liposomal membranes and to transfer more rapidly to cells than purified carboxyfluorescein (Ralston et al., 1981). In line with this it has been found that liposomes prepared with unfractionated AlSPc were very sticky and bound to cells non-specifically, thus decreasing the differential toxicity between targeted cells and untargeted controls. This effect was slightly reduced by first passing the dye down an LH 20 column which removed the most lipophilic components such as the di, mono and unsulphonated phthalocyanine molecules (personal observations). For this reason the most polar fraction of AlSPc was selected for encapsulation in liposomes. Another source of non-specific interaction of AlSPc and cells lies in its avid binding to protein (Ben-Hur & Rosenthal, 1985*a*,*b*; Chan et al., 1986; personal observations). This avidity would pose problems were AlSPc to be bound directly to a monoclonal antibody. We have observed (for example) 5–10% mole for mole non-specific binding of AlSPc to human serum albumin, depending on pH (7–9). Sequestration of AlSPc in liposomes has the potential for preventing it binding non-specifically to proteins present in the medium and on the surface of cells. Furthermore, liposome delivery allows a larger number of AlSPc molecules to be directed to each binding site on the cell. Roberts et al. (1987) coupled only two porphyrin molecules per antibody molecule, whereas our liposomes contained an average of 53 molecules of AlSPc, an amplification of more than 25 times.

The results of this paper show that AlSPc can be successfully encapsulated in liposomes and targeted against cells without non-specific phototoxicity, and that the degree of toxicity is dependent on the dose of both light and photosensitiser. The treatment is rapid and requires only 45 min for antibody binding plus up to 25 min for light treatment. In any targeting system there is the possibility of unwanted non-specific effects. These have been minimised in the present system by a number of factors. Firstly the liposomes are reasonably stable (20% leakage of contents after 4 months storage at 4°C) so there is little leakage into the medium. Secondly, once the light source is removed there is no further activation of the dye and any cell damage will be due to the effects of the toxic species already generated. Thirdly, even if some dye does escape from liposomes, this fraction of AlSPc which has been selected is not easily taken up into cells due to its polar nature. No light mediated toxicity was produced in 791T cells by free AlSPc even at ten-fold the concentration used in targeted liposomes (Figure 1).

For effective cytotoxicity, previous classes of drugs have needed to gain access to the cell interior, whether presented in liposomes (e.g. methotrexate) or attached to the antibody as an immunotoxin (e.g. ricin). The Ag/Ab/drug complex must be endocytosed for effective action. However, not all antigens or receptors are internalised and some are shed from the surface and are thus unsuitable for drug delivery.

AlSPc and other photosensitisers, for example chlorin $e_6$ (Oseroff *et al.*, 1986) do not necessarily need internalisation to be effective cytotoxic agents. In the present experiments conditions were used (incubtion at 4°C) that would not favour the kinetics of endocytosis or antigen shedding; therefore AlSPc would not gain access to the interior of the cell. The effect of targeting the AlSPc in liposomes therefore seems to be to concentrate the photosensitiser in sufficient proximity to the cell for it to have a cytotoxic action after light activation. It is possible that the liposome membrane is destroyed in the process. The main target sites for the active entity are probably the cell membrane and nearby cytoplasmic components which are attacked by singlet oxygen after its generation and diffusion from the lysed liposome. Singlet oxygen causes damage to cells by oxidation of biological molecules important for the integrity and functioning of the cells such as phospholipids, cholesterol, amino acids and nucleic acids (Grossweiner, 1981). The postulated mechanism of action is thus similar to the lytic action of the complement system on the integrity of the cell membrane, and therefore, unlike many cytotoxic drugs AlSPc is able to destroy resting or slow growing cancer cells as well as actively dividing cells, which suggests it has the potential of a potent anticancer agent.

The phototoxic action of AlSPc is very rapid compared with drugs which must be internalised to cause toxicity. After 4 h incubation of 791T cells at 37°C only 30% of 791T/36 on the surface is internalised under saturating conditions (Huehns, 1986). Any drug attached to the antibody is likely to take a similar time to gain access to the cell. By comparison, production of free radicals on illumination of 791T/36 bound liposomes containing AlSPc is practically instantaneous.

It is uncertain to what degree singlet oxygen will be able to damage adjacent cells. The mean distance a singlet oxygen molecule can diffuse during its lifetime has been quoted as 1,000 Å (Lindig & Rodgers, 1981; Grossweiner, 1981). It has been suggested that this is too short a distance for diffusion to neighbouring cells (Yemul *et al.*, 1987), though that would depend on how close the cells are positioned. Certainly in the present experiments the conditions are such that the cells are remote from each other and there is little possibility of singlet oxygen diffusion from one cell to another. Furthermore, Sonada *et al.* (1987) provide evidence that exogenous AlSPc is not responsible for haemolysis of red blood cells, presumably because the active singlet oxygen was not capable of diffusing close enough at sufficient concentration, while bound AlSPc haemolysed the cells. However, the opposite was suggested by Oseroff *et al.* (1986), that the diffusion distance of singlet oxygen may be sufficient to cause destruction of adjacent cells, and that shed complexes may still be effective provided they remain close. Our experiments confirm that AlSPc in liposomes bound to cells by antibody causes phototoxicity while free AlSPc does not.

In the present experiments a monoclonal antibody is used to target liposomes directly to the cells. However, for some cell types, perhaps expressing low density antigens, a mixture of such antibodies rather than a single one might be more suitable for attaining maximum cell kill. In this situation an indirect method of targeting is envisaged, in which cocktails of mouse monoclonal antibodies attach to the cells and the phototoxic liposome is coupled to polyclonal anti-mouse antibody which will bind to all the monoclonal antibodies. Previously, protein A was used for indirect targeting of liposomes as described by Gray *et al.* (1988), but use of an anti-mouse antibody would be more versatile since it ensures binding to all Ig subclasses whereas protein A has restricted binding at physiological pH. Of course, where rat or any other species of monoclonal antibody are used the appropriate anti-species antibody must be attached to the liposome.

Most drug-Ab conjugates or liposome-Ab targeted drugs have had reasonable success as cytotoxic agents *in vitro*, but not *in vivo*, due to rapid uptake by the reticulo endothelial system, which prevents the drug reaching the target (Weinstein, 1984), though some success has been achieved by exploiting the uptake of phthalocyanine containing liposomes by low density lipoproteins (LDL), and their subsequent delivery to tumour tissues with high LDL receptor expression (Reddi *et al.*, 1987). However, in some situations, such as treatment of malignancies affecting the body cavities, for example ovarian carcinoma, where *ex vivo* manipulations of cells are involved such as bone marrow transplantation, or where the affected organ is accessible to instillation, such as the bladder, they can still have an important role to play. This is particularly so in conditions where current treatment is inadequate, such as advanced ovarian carcinoma, or in autologous bone marrow transplantation (ABMT) where it would be used for purging as, for example, in multiple myeloma.

Intraperitoneal treatment of ovarian carcinoma has been attempted with limited success by administration of chemotherapeutic agents at much higher concentrations than would be given systemically (Richardson *et al.*, 1985). An advantage of such treatment is lower systemic toxicity, but there are local side effects due to free cytotoxic drug. With our liposome system tumour cells would be targeted by monoclonal antibodies and the normal peritoneum would be protected from non-specific uptake by the insulating effects of the encapsulating lipid. Intraperitoneal photodynamic therapy (PDT) of ovarian cancer would be feasible by i.p. injection of Ab targeted AlSPc liposomes followed by laser light delivered by quartz fibre for photoactivation.

This technique may also be widely applicable for the *ex vivo* purging of residual disease from the bone marrow of patients undergoing ABMT after high dose chemotherapy or total body irradiation to eliminate bulk disease. Pharmacological agents have been used to purge marrow from patients with lymphoma and leukaemia, but they lack selectivity and are equally toxic to normal haemopoietic cells (Singer & Linch, 1987). With the right combination of monoclonal antibodies it should be possible to target and destroy tumour cells in bone marrow while sparing the normal haemopoietic precursor cells necessary for re-engraftment.

Purging is not thought to be necessary for ABMT where bulk disease can be satisfactorily eliminated and there is no detectable disease in the marrow. However, where there is marked infiltration of tumour cells in the bone marrow, as in multiple myeloma, then purging with antibody targeted AlSPc liposomes might offer a better chance of recovery without relapse.

Finally, it has been shown that monoclonal antibodies (produced against antigens of human bladder transitional cell carcinoma) bind preferentially to tumour rather than normal mucosa after intravesical injection (Chopin & deKernion, 1986). Targeting AlSPc-liposomes with such antibodies could provide an alternative route for treatment of bladder tumours.

Thanks to Russel Svenson of the Royal Institution, London for advice on the purification of AlSPc and for running the HPLC. This work was supported by a grant from Bloomsbury Health Authority.

**References**

BARBET, J., MACHY, P. & LESERMAN, L.D. (1981). Monoclonal antibody covalently coupled to liposomes: specific targeting to cells. *J. Supramol. Struct. Cell. Biochem.*, **16**, 237.

BEN-HUR, E. & ROSENTHAL, L. (1985*a*). Factors affecting the photokilling of cultured Chinese hamster cells by phthalocyanines. *Radiat. Res.*, **103**, 403.

BEN-HUR, E. & ROSENTHAL, L. (1985*b*). Photosensitised inactivation of Chinese hamster cells by phthalocyanines. *Photochem. Photobiol.*, **42,** 129.

BRASSEUR, N., ALI, H., AUTENTIETH, D., LANGLOIS, R. & VAN LIER, J.E. (1985). Biological activities of Phthalocyanines-111. Photoinactivation of V-79 Chinese hamster cells by tetrasulphophthalocyanines. *Photochem. Photobiol.*, **42,** 515.

CHAN, W.S., SVENSON, R., PHILLIPS, D. & HART, L.R. (1986).Cell uptake, distribution and response to aluminium chlorosulphonated phthalocyanine, a potential anti-tumour photosensitiser. *Br. J. Cancer*, **53,** 255.

CHOPIN, D.K. & DEKERNION, J.B. (1986). Detection of transitional cell carcinoma in bladder by intravesical injection of monoclonal antibodies. *Urol. Res.*, **14,** 145.

EMBLETON, M.J., GUNN, B., BYERS, V.S. & BALDWIN, R.W. (1981). Antitumour reactions of monoclonal antibody against a human osteogenic-sarcoma cell line. *Br. J, Cancer*, **43,** 582.

GARNETT, M.C., EMBLETON, M.J., JACOBS, E. & BALDWIN, R.W. (1983). Preparation and properties of a drug-carrier-antibody conjugate showing selective antibody-directed cytotoxicity *in vitro. Int. J. Cancer*, **31,** 661.

GRAY, A.G., MORGAN, J., LINCH, D.C. & HUEHNS, E.R. (1988). Uptake of antibody directed cytotoxic liposomes by CD3 on human T-cells. *Clin. Exp. Immunol.*, **72,** 168.

GROSSWEINER, L.I. (1981). Photosensitisation of biomolecules *in vivo*. In *Oxygen and Oxy-radicals in Chemistry and Biology*, Rodgers, M.A.J. & Powers, E.L. (eds) p. 479. Academic Press: London.

HUEHNS, T.H.A.Y. (1986). Antibody directed drug conjugates. B.Med.Sc. degree thesis, University of Nottingham.

LINDIG, B.A. & RODGERS, A.J. (1981). Rate parameters for the quenching of singlet oxygen by water-soluble and lipid soluble substrates in aqueous and micellar systems. *Photochem. Photobiol.*, **33,** 627.

MACHY, P., BARBET, J. & LESERMAN, L.D. (1982). Differential endocytosis of T and B lymphocyte surface molecules evaluated with antibody bearing liposomes containing methotrexate. *Proc. Natl Acad. Sci. USA*, **79,** 4148.

OSEROFF, A.R., OHUOHA, D., HASAN, T., BOMMER, J.C. & YARMUSH, M.L. (1986). Antibody-targeted photolysis: selective photodestruction of human T-cell leukaemia cells using monoclonal antibody-chlorin $e_6$ conjugates. *Proc. Natl Acad. Sci. USA*, **83,** 8744.

PAQUETTE, B., ALI, H., LANGLOIS, R. & VAN LIER, J.E. (1988). Biological activities of phthalocyanines-V111. Cellular distribution in V-79 Chinese hamster cells and phototoxicity of selectively sulphonated aluminium phthalocyanines. *Photochem. Photobiol.*, **47,** 215.

PERKINS, A.C., PIMM, M.V. & BIRCH, M.K. (1985). The preparation of characterisation of $^{111}$In-labelled 791T/36 monoclonal antibody for tumour immunoscintigraphy. *Eur. J. Nucl. Med.*, **10,** 296.

RALSTON, E., HJELMELAND, L.M., KLAUSNER, R.D., WEINSTEIN, J.N. & BLUMENTHAL, R. (1981). Carboxyfluorescein as a probe for liposome-cell interactions, effect of impurities and purification of the dye. *Biochim. Biophys. Acta*, **649,** 133.

REDDI, E., LO CASTRO, G., BIOLO, R. & JORI, G. (1987). Pharmacokinetic studies with zinc(11)phthalocyanine in tumour bearing mice. *Br. J. Cancer*, **56,** 597.

RICHARDSON, G.S., SCULLY, R.E., NIKRUI, N. & NELSON, J.H. (1985). Common epithelial cancer of the ovary. Part 2. *N. Engl. J. Med.*, **312,** 474.

ROBERTS, C.J., FIGARD, S.D., MERCER-SMITH, J.A., SVITRA, Z.V., ANDERSON, W.L. & LAVALLEE, D.K. (1987). Preparation and characterisation of copper-67 porphyrin-antibody conjugates. *J. Immunol. Methods*, **105,** 153.

ROE, R., ROBINS, R.A., LAXTON, R.R. & BALDWIN, R.W. (1985). Kinetics of divalent monoclonal antibody. Binding to tumour cell surface antigens using flow cytometry: standardisation and mathematical analysis. *Mol. Immunol.*, **22,** 11.

ROUSSEAU, J., AUTENRIETH, D. & VAN LIER, J.E. (1983). Synthesis, tissue distribution and tumour uptake of $^{99}$Tc tetrasulphophthalocyanine. *Int. J. Appl. Radiat. Isotopes*, **34,** 571.

SINGER, C.R.J. & LINCH, D.C. (1987). Comparison of the sensitivity of normal and leukaemic myeloid progenitors to *in vitro* incubation with cytotoxic drugs: a study of pharmacological purging *Leuk, Res.*, **11,** 953.

SINGER, C.R.J., BOWN, S.G., LINCH, D.C., HUEHNS, E.R. & GOLDSTONE, A.H. (1987). Phthalocyanine photosensitisation for *in vitro* elimination of residual acute non-lymphoblastic leukaemia: preliminary evaluation. *Photochem. Photobiol.*, **46,** 745.

SINGER, C.R.J., LINCH, D.C., BOWN, S.G., HUEHNS, E.R. & GOLDSTONE, A.H. (1988). Differential phthalocyanine photosensitisation of acute myeloblastic leukaemia propgenitor cells: a potential purging technique for autologous bone marrow transplantation. *Br. J. Haematol.*, **68,** 417.

SPIKES, J.D. (1986). Phthalocyanines as photosensitisers in biological systems and for the photodynamic therapy of tumours. *Photochem. Photobiol.*, **43,** 691.

SONODA, M., KRISHNA, C.M. & RIESZ, P. (1987). The role of singlet oxygen in the photohemolysis of red blood cells sensitised by phthalocyanine sulphonates. *Photochem. Photobiol.*, **46,** 625.

STEWART, J.C.M. (1980). Colorimetric determination of phospholipids with ammonium ferrothiocyanate. *Anal. Biochem.*, **104,** 10.

TRALAU, C.J., BARR, H., SANDEMAN, D.R., BARTON, T., LEWIN, M.R. & BOWN, S.G (1987). Aluminium sulphonated phthalocyanine distribution in rodent tumours of the colon, brain and pancreas. *Photochem. Photobiol.*, **46,** 777.

WEINSTEIN, J.N. (1984). Liposomes as drug carriers in cancer therapy. *Cancer Treat. Rep.*, **68,** 127.

WILSCHUT, J. (1982). Preparation and properties of phospholipid vesicles. In *Lipsome Methodology*, Leserman, L.D. & Barbet, J. (eds) p. 9. INSERM: Paris.

YEMUL, S., BERGER, C., ESTABROOK, A., SUAREZ, S., EDELSON, R. & BAYLEY, H. (1987). Selective killing of T-lymphocytes by phototoxic liposomes. *Proc. Natl Acad. Sci. USA*, **84,** 246.